# Leadership position and physician visits – results of a nationally representative longitudinal study in Germany

**DOI:** 10.1186/s12995-018-0216-7

**Published:** 2018-10-25

**Authors:** Katrin Christiane Reber, Hans-Helmut König, André Hajek

**Affiliations:** Department of Health Economics and Health Services Research, University Medical Center Hamburg-Eppendorf, Hamburg Center for Health Economics, Hamburg, Germany

**Keywords:** Leadership position, Health care utilization, Longitudinal studies, Germany

## Abstract

**Background:**

So far, studies within the occupational field have largely concentrated on working conditions and job stressors and staff members’ or subordinate health. Only a few have focused on managers in this context, but studies are missing that explicitly look at the relation between leadership position and health care use (HCU). Thus, the purpose of this study was to examine the potential effects of a change in leadership position on HCU in women and men longitudinally.

**Methods:**

Data were drawn from a nationally representative longitudinal study in Germany (German Socio-Economic Panel, GSOEP). Data from 2009 and 2013 were used. Leadership position was divided into (i) top management, (ii) middle management, (iii) lower management, and (iv) a highly qualified specialist position. The number of physician visits in the preceding 3 months were used to quantify HCU (*n* = 2140 observations in regression analysis; 69% male).

**Results:**

Adjusting for various potential confounders (e.g., age, self-rated health, chronic conditions, and personality factors), Poisson FE regression analysis revealed that changes from a highly qualified specialist position to the top management were associated with a *decrease* in the number of physician visits in men (β = .47, *p* < .05), but not in women. Gender differences (gender x leadership position) were significant.

**Conclusions:**

Findings of this study emphasize the impact of leadership positions on the number of physician visits in men. Further study is required to elucidate the underlying mechanisms.

## Background

There is unequivocal evidence that socioeconomic position, commonly measured by occupational class, education or income, is a leading determinant of health. Lower socioeconomic positions have generally been linked to unhealthier behaviors compared to higher positions [1]. Several studies have shown that health and health–related outcomes vary considerably by occupation [2, 3]. Occupational rank/position and employment conditions were identified as important factors in creating these health differentials [4]. Occupational position has been reported to be associated with both physical and psychological health and the association between job status and health appeared to be quite robust across different countries and settings and after adjusting for other socioeconomic position measures like education or income (though these measures will be interrelated [5, 6]) [7]. There is further evidence that the magnitude of the association between occupational position and health differs between men and women. In women, the relationship between occupational position and self-perceived health was less pronounced than in men [8]. These gender differences in the prevalence of unfavorable physical and mental health outcomes have been at least partly attributed to gender discrimination in the labor market [9–11].

In addition to inequalities in health outcomes between different socioeconomic groups as well as between men and women, numerous studies have shown that socioeconomic position affects use of health care services. Findings from Germany and other European countries indicated a general tendency toward higher health services use among lower socioeconomic groups [12–14]. Furthermore, studies generally assumed a directionality from work to health or use of health care services, and not vice versa [15, 16].

Previous research has also suggested that changes in occupational position may affect an individual’s health status. For example, Halleröd and Gustafsson [17] found a link between changes in occupational prestige and changes in morbidity such that a more prestigious career development resulted in more favorable health outcomes. Both short- and long-term negative health consequences of experienced or anticipated job change have also been confirmed by other studies [18, 19]. For example, poorer self-rated health and an increased risk of minor psychiatric disorders have been reported by white collar civil service employees when compared to those not affected by job change [18, 19]. However, these effects were found to be different for men and women and to depend on occupational grade. In particular, men and women in the highest employment grade as well as men in the middle grade reported significantly more psychiatric disorders and poorer health [18]. Though contrary to previous suggestions that negative health effects are more common among persons of lower income positions [20], also another study found health effects and the risk of sick leave to be greater amongst higher income positions [21]. Poorer health status among higher grade employees / managers could thus lead to increased health care use (HCU). However, since higher positions generally come along with greater responsibilities and workload, less time or other factors e.g., fear of loss of power (an increase in the power of subordinates might reduce their own [22]) may prevent them from using health care services. As regards gender differences in the context of change in leadership position and HCU knowledge is limited. Inconclusive results have been found when investigating gender-specific health effects in times of organizational change [18, 23]. Yet, it has been noted that gender bias may be aggravated during phases of organizational and job change and this bias is possibly more obvious at positions that are higher in hierarchy [24] - not only in terms of hiring bias but also in terms of job promotion or career progression as well as in terms of destabilization of professional careers in times of organizational change [25].

While the relation between socio-economic position and HCU has received quite some attention, there is little research investigating the association between occupational position and HCU when employed in similar circumstances, experiencing a similar work or job “role”, i.e., leadership position.

So far, studies within the occupational field have largely concentrated on working conditions and job stressors and staff members’ or subordinate health [26]. Only a few have focused on managers in this context [27, 28] but studies are missing that explicitly look at the relation between leadership position and HCU. However, the health of an organization’s leader /manager is of crucial importance for the leader, for the organization and for its staff, because managers’ poor health can negatively affect both team and an organization’s performance [28, 29]. Consequently, based on a large nationally representative sample, the purpose of this study was to examine whether changes in leadership position (e.g., from middle management to the top management) are associated with changes in HCU (i.e., physician visits) among women and men using a longitudinal approach.

We hypothesize that a change to a higher leadership position is accompanied by more responsibilities, higher workload, but also more power and prestige. On the one hand, we hypothesize that more responsibilities and higher workload result in higher stress or poorer health status and thus increase physician visits. On the other hand, we hypothesize that prestigious positions are accompanied by less time for physician visits. In addition, individuals in these leadership positions might have better coping strategies to handle potential health problems or job stress [30].

There are well-known gender differences in socioeconomic position, particularly in occupational position and due to extensive evidence that gender matters in HCU – as reported in a recent systematic review [31]. For example, based on American or Australian samples it has been shown that women were more likely to consult a physician [32, 33]. Consequently, we conducted analyses separately for women and men. It was further tested whether gender moderates the impact of the leadership position on the number of physician visits.

## Methods

### Sample

Data were drawn from the German Socio-Economic Panel (GSOEP), located at the German Institute for Economic Research (DIW Berlin), beginning in 1984. It is a nationally representative survey of the German population. Above 20,000 individuals (about 11,000 households) were interviewed on an annual basis. A very broad range of topics is covered in the GSOEP such as occupational status, subjective well-being, health or attitudes. It has been shown that survey attrition is low and re-interview response rates are very high in the GSOEP [34, 35]. For further details concerning the GSOEP (e.g., sample composition or subsamples), please see Wagner et al. [36]. In the present study, data from two waves (2009 and 2013) were used for reasons of data availability. Thus, mid-term associations between changes in leadership positions and HCU were analyzed. In other words: We restricted our sample to individuals who changed their leadership position from 2009 and 2013.

### Outcome measure: Physician visits

The self-reported number of physician visits in the preceding 3 months was used to measure the number of outpatient physician visits.

### Independent variables

Based on the behavioral model developed by Andersen et al. [37], explanatory variables were selected. The Andersen model distinguishes between predisposing characteristics (e.g., sex and age), enabling resources (e.g., income) and need factors (e.g., self-rated health or chronic illnesses).

For *predisposing characteristics*, age, gender family status, and the kinds of leadership position were included.

The self-reported kinds of leadership position were divided into

- Top management (for example, executive board, business director, division manager)
- Middle management (for example, department head, regional director)
- Lower management (for example, group supervisor, section head, management of a small branch office / small business)
- Highly qualified specialist position (for example, project head)

We note that we only analyzed individuals who fell in one of the above categories. Changes in the leadership position from 2009 to 2013 were analyzed.

Family status was dichotomized into those married or living together with a partner and those not living with a partner, i.e. divorced, widowed or single. As regards *need factors*, self-rated health (1 = “bad” and 5 = “very good”) and a count score of chronic conditions was used (diabetes, asthma, cardiac disease, cancer, heart attack, migraine, high blood pressure and dementia). The self-rated health single item measure has widely been used in previous studies [38].

There are some personality traits which literature has well documented to be related to leadership position (e.g. extraversion). Moreover, evidence exists showing that personality traits affect physical and mental health, and play a role in pursuing (un)healthy behaviors and in achieving (un)favorable health outcomes [39, 40]. Studies have further shown that personality (e.g., neuroticism) is important in HCU [41–43]. Personality is commonly divided into five big traits [44]. These big traits are agreeableness, conscientiousness, extraversion, neuroticism and openness to experience. Agreeableness refers to the tendency to get along well with others. Conscientiousness refers to the tendency to be well organized. Extraversion refers to the tendency to be talkative or sociable. Neuroticism refers to the tendency to be insecure or anxious. Openness to experience refers to the tendency to take risks or to be imaginative. In the GSOEP, the short version of the Big Five Inventory (BFI-S) was used. Three items per dimension were used. Each item was rated on a seven-point Likert scale ranging from 1 = “does not apply to me at all” to 7 = “applies to me perfectly”. It has been demonstrated that the BFI-S has satisfactory psychometric properties [45]. While those traits have predominantly been considered to remain stable over time, more recent findings point to the dynamic effects of personality change and their implications for the personality-health link [39, 46]. Therefore, these factors were included in regression analysis as time-varying variables.

### Statistical analysis

First, descriptive statistics for the analytical sample were computed. Second, panel regression models were used to assess the longitudinal association between change in leadership positions and HCU, adjusting for potential confounders (age, marital status, self-rated health, number of chronic diseases, and personality traits).

In large survey studies, unobserved heterogeneity (time-constant unobserved factors such as genetic disposition) is a key problem. For example, it is almost impossible to control for differences between individuals in genetic factors in these studies [47]. This is a critical problem when these unobserved factors are systematically correlated with the explanatory variables. The reason is that most of the widely used panel regression models such as random effects regressions produce inconsistent estimates when this correlation is present. Or, to put it another way: These panel regression models rest on the assumption of no correlation between the explanatory variables and the time-constant unobserved factors [48]. In contrast to these panel regression models, FE regression produce consistent estimates even if this strong assumption is violated. Thus, FE regressions were used in the present study. This choice was supported by a Hausman-test [49] – the Hausman test statistic was statistically significant, with *p* < .001. This test indicated that the effects are associated with the explanatory variables and thus the RE model cannot be estimated consistently.

FE regressions (“Within-estimator”) only exploit transitions within individuals over time. Hence, the results can be interpreted as “Average Treatment Effect on the Treated” (ATET) [47]. In other words: Our findings are not generalizable to the whole population.

For example, it is worth emphasizing that changes from a “highly qualified specialist position” to “lower management” within an individual over time were examined in our study. Factors constant within individuals over time such as gender can only be included as moderating variables (e.g., gender x leadership position).

Due to power issues in our FE regression analysis changes in both directions were covered, i.e., changes from lower level leadership positions to higher level leadership positions as well as changes from higher level leadership positions to lower level leadership positions.

In the current study, cluster robust standard errors were computed [50]. A *P* value less than 0.05 was deemed statistically significant. Analyses were conducted using Stata 15.0 (Stata Corp., College Station, Texas).

In sensitivity analysis, satisfaction with free time, family life, and job (if employed) (each variables ranges from 0 = ‘totally unhappy’ to 10 = ‘totally happy’) were added to the main model. In further sensitivity analysis, concerns about the job security (if employed) (1 = very concerned; 2 = somewhat concerned; 3 = not concerned at all) and the difficulty of finding an appropriate position (“If you were currently looking for a new job: Is it or would it be easy, difficult, or almost impossible to find an appropriate position?”; 1 = easy; 2 = difficult; 3 = “almost impossible”) were added to the main model. In other sensitivity analyses, (log) equivalence income and working hours per week were added to our main model. In another robustness check, negative binomial fixed effects (FE) regressions were used.

## Results

### Sample characteristics

Pooled sample characteristics for individuals included in FE regression analysis with physician visits in the past 3 months as outcome variable are depicted in Table 1 (stratified by gender, men: 1476 observations; women: 664 observations).

**Table 1:** Sample characteristics for individuals included in fixed effects regressions (Wave 2009 and 2013, pooled; 2140 observations)

	Men (1476 observations)		Women (664 observations)	
	N/Mean	%/SD	N/Mean	%/SD
Age (in years)	48.3	9.4	46.2	9.3
Married, living together with spouse	372	25.2%	288	43.4%
Self-rated health (from 1 = “very good” to 5 = “bad”)	2.5	0.8	2.5	0.8
Number of chronic diseases	0.4	0.7	0.5	0.7
Number of chronic diseases: 0	944	64.0%	422	63.5%
Number of chronic diseases: 1	420	28.4%	182	27.4%
Number of chronic diseases: 2	94	6.4%	43	6.5%
Number of chronic diseases: 3	16	1.1%	16	2.4%
Number of chronic diseases: > 3	2	0.1%	1	0.2%
- Agreeableness	15.4	2.9	16.0	2.9
- Conscientiousness	17.6	2.5	18.2	2.4
- Extraversion	14.5	3.3	15.4	3.3
- Openness to experience	13.6	3.3	14.2	3.5
- Neuroticism	10.1	3.4	11.3	3.5
Physician visits in the preceding 3 months	2.1	3.2	2.5	3.0
Leadership position				
Top management	319	21.6%	91	13.7%
Middle management	416	28.2%	161	24.3%
Lower management	477	32.3%	283	42.6%
Highly qualified specialist position	264	17.9%	129	19.4%

About two-thirds were male. The average age for men was 48.3 years, and for women, the average age was 46.2 years. While in men approximately one half were in the middle or top management, less than 40% held these positions in women. In men, the average number of physician visits in the past 3 months equaled 2.1, the average number was 2.5 in women. Further details are provided in Table 1.

It is worth noting that the average number of doctor visits (GP and specialist visits) is about 8.5 among the adult population in Germany per year [51].

### Regression analysis

Results of Poisson FE regressions are depicted in Table 2. In Table 2, Poisson coefficients with cluster-robust standard errors were reported. In total, 390 intraindividual changes in leadership positions were used in FE regression analysis. Changes from a ‘highly qualified specialist position’ to ‘top management’ from 2009 to 2013 were associated with a decrease in the number of physician visits in the preceding 3 months in men (β = .47, *p* < .05), but not in women (with significant gender differences: *p* = .008).

**Table 2:** Determinants of physician visits in the past three months. Results of FE poisson regressions

Independent variables	(1)	(2)	(3)	(4)
	Doctor visits – Total sample	Doctor visits - Men	Doctor visits - Women	Doctor visits – with interaction
Age (in years)	−0.01	− 0.00	− 0.04*	− 0.02
	(0.01)	(0.01)	(0.02)	(0.01)
Other marital statuses (Ref.: Married, living together with spouse)	−0.15	− 0.02	− 0.40*	− 0.16
	(0.14)	(0.19)	(0.16)	(0.14)
Self-rated health (from ‘very good’ to ‘bad’)	0.52***	0.55***	0.43***	0.51***
	(0.05)	(0.07)	(0.08)	(0.05)
Number of chronic diseases	0.16**	0.14+	0.22+	0.17**
	(0.06)	(0.07)	(0.12)	(0.06)
Neuroticism (higher values indicate higher neuroticsm)	0.01	0.01	0.00	0.01
	(0.01)	(0.02)	(0.02)	(0.01)
Extraversion (higher values indicate higher extraversion)	−0.02	−0.01	−0.03	−0.01
	(0.02)	(0.02)	(0.03)	(0.02)
Openness to experience (higher values indicate higher openness)	−0.00	0.01	−0.01	−0.00
	(0.01)	(0.02)	(0.02)	(0.01)
Agreeableness (higher values indicate higher agreeableness)	−0.01	0.00	−0.04	− 0.01
	(0.02)	(0.02)	(0.03)	(0.02)
Conscientiousness (higher values indicate higher conscientiousness)	0.01	0.02	0.01	0.01
	(0.02)	(0.03)	(0.02)	(0.02)
Leadership position: - Middle management (Ref.: Top Management)	0.14	0.22	−0.03	0.23
	(0.16)	(0.20)	(0.28)	(0.20)
- Lower management	0.15	0.41+	−0.36	0.43*
	(0.16)	(0.22)	(0.23)	(0.21)
- Highly qualified specialist position	0.25	0.47*	−0.24	0.49*
	(0.18)	(0.22)	(0.31)	(0.21)
Gender (Ref.: male) x Middle management				−0.32
				(0.34)
Gender (Ref.: male) x Lower management				−0.83**
				(0.31)
Gender (Ref.: male) Highly qualified specialist position				−0.78*
				(0.38)
Observations	2140	1476	664	2140
Number of Individuals	1070	738	332	1070

First column: total sample; second column: men; third column: women; fourth column: total sample, with interaction term gender x leadership position; Poisson coefficients were reported; cluster-robust standard errors in parentheses

*** *p* < 0.001; ** *p* < 0.01; * *p* < 0.05; + *p* < 0.10

While worsening self-rated health was associated with an increase in the outcome measure in the total sample and in both genders, an increase in the number of chronic diseases was only associated with an increase in the outcome measure in the total sample. Moreover, increasing age and changes from ‘married’ to another marital status were associated with a decrease in the outcome measure in women, but not in men. None of the personality factors reached statistical significance.

### Sensitivity analysis

In sensitivity analysis (results of sensitivity analysis are not shown, but are available upon request), satisfaction with (i) free time, (ii) family life and (iii) job were added to the main model. However, findings with regard to the leadership position remained virtually the same. In further sensitivity analysis, concerns about the job security and the difficulty of finding an appropriate position were added to the main model. In another robustness check, it was additionally adjusted for income. In additional sensitivity analysis, it was adjusted for working hours per week. Again, our results remained almost the same.

Moreover, robustness was checked by using negative binomial FE regression models. In terms of significance, the relation between leadership position and physician visits was nearly identical.

## Discussion

### Main findings

The aim of the present study was to investigate the association between leadership position and HCU in women and men longitudinally. Adjusting for various potential confounders such as self-rated health, FE regression analysis revealed that changes from a highly qualified specialist position to the top management were associated with a *decrease* in the number of physician visits in men, but not in women. Gender differences (gender x kind of leadership) achieved statistical significance.

### Possible explanations of how (change in) leadership position contributes to healthcare use

Inconsistent findings have been reported regarding the relationship between socioeconomic status and healthcare use (outpatient and inpatient) in Germany based on cross-sectional studies [13, 14]. However, these cross-sectional findings are not directly comparable to ours as we consider leadership position and not socioeconomic status in general as explanatory variable using a specific sample of the German labor force as well as a longitudinal approach. In addition, we specifically examined physician visits and not general healthcare use as outcome measure. More generally, studies are missing which explicitly focus on the relation between *leadership position* and healthcare use both cross-sectionally and longitudinally. In conclusion, our findings are difficult to compare with previous studies.

As regards possible explanations, it could be that a change in leadership is accompanied by a change in one’s own perceptions of discomfort or chronic conditions [52]. Due to workload and time constraints, symptoms may be ignored. This change in perception might cause the decrease in physician visits. Moreover, it might be that the change in leadership position (from lower to higher) heavily restricts managers’ time available, e.g., to use health care services, and therefore physician visits will decrease [53]. For example, in our analytical sample, the lower the leadership position is, the higher is the leisure time in hours (association between leisure time in hours and leadership position (from 1 = top management to 4 = highly qualified specialist position): Spearman’s rho = .08, *p* < .001) and the lower are the working hours (association between working hours per week and leadership position: Spearman’s rho = −.28, *p* < .001).

High-status professionals may also have more resources available to buffer the potentially negative impact of work stress. It has been previously suggested that individuals in higher occupational positions feel less burdened by high job stress compared to those in lower occupational positions [54, 55]. Possibly, the change to (higher) leadership position in *men* is associated with an increased engagement in health behaviors (e.g., physical activity or healthy diet) to cope with increased stress levels in the leadership position [56]. Thus, their health status will be less affected and consequently, the change in leadership position in men might lead to decreases in physician visits. However, the change to a higher position frequently comes along not only with more responsibilities but also higher stakes and increased level of work stress. Therefore, self-selection is likely to play a role; and individuals who consider themselves suitable and able to tolerate high levels of stress may be more likely to move into higher management positions [57].

Another explanation could be that individuals in top management positions may have access to a broader network through which they may get more social support [58] which could positively influence health. Changes to higher leadership positions may also come along with positive feelings of appreciation and thus higher levels of job satisfaction [59]. This may eventually result in reduced HCU. However, our findings remained almost the same, when we included various types of satisfaction (i.e., job, family, leisure time) in sensitivity analysis.

Possibly, higher-rank managers expect a tenured, more secure position and hence will be more involved in their jobs [60]. At the same time they may face higher job demands and responsibilities but also more competitive pressure within these ranks. This could translate in greater fear of job degradation and thus result in fewer physician visits (for example to avoid absence from work due to illness). In a similar vein, higher-level managers may feel more committed to the organization and thus could be more inclined to sacrifice own interests and needs for the good of the organization. Consequently, they may use physician services less.

Several speculative explanations are possible why changes in leadership positions were not associated with HCU in *women*. First, there might be heterogeneous effects in female managers. While changes from the lower management to the top management might be associated with an *increase* in HCU among some women who score high in prudence (for example, to avoid negative health effects on their children or family), it might *decrease* HCU in other women who score high in competitiveness. Future studies are needed to clarify this issue.

While one may argue that the combinations of career and family obligations could lead to more HCU in women compared to men, research found that multiple roles and a challenging job may buffer against stress and entail positive health effects [61–63]. As a result, these women may not need physician services and decrease their use. However, future studies are required to investigate this in depth. Another more general explanation might be that women’s health care use is typically affected by need factors rather than external circumstances [31].

Furthermore, the non-significant association might be explained by a lack of statistical power (small number of changes in leadership positions among women), but the number is increasing steadily [64]. Particularly in women, a hiring bias may still be present despite advancement of equality between genders in leadership positions [24]. Yet, it could also be that women have different preferences and are less likely to opt for these positions compared to their male counterparts due to double burden of family and working life [65]. Thus, future research with greater statistical power is required.

While personality traits have been suggested to predict health outcomes [46], this could not be confirmed in our study for HCU. This might be explained by a lack of statistical power. Moreover, a recent study showed that only high neuroticism was associated with HCU among Dutch young adults [66], whereas the other personality factors were not associated with high GP costs (dichotomized outcome measure with low and high GP costs). However, we expect that significant associations between personality factors and HCU (particularly with mental HCU [67]) might appear in a large sample representing the general population [41].

### Strengths and limitations

To the best of our knowledge, this is the first study examining the association between leadership position and HCU in women and men longitudinally. Data were drawn from a nationally representative sample. Four kinds of leadership positions were used. In addition, one of the main challenges in large survey studies - the problem of unobserved heterogeneity - was reduced using FE regressions. In accordance with recommendations [68] and in line with previous studies investigating the determinants of HCU [31], a short recall period (3 months) was used for physician visits. Consequently, we assume that the recall bias was small and many health-related events were covered in the current study. Panel attrition is a common source of bias in longitudinal studies. However, it has been demonstrated that panel attrition is only a minor problem in the GSOEP [34]. Leadership position was based on self-reports on a scale specifically developed for the SOEP (personal communication), which is a potential limitation.

While we cannot dismiss the possibility of an endogeneity bias (physician visits affect leadership position), we strongly believe that this is rarely the case. Moreover, findings from other longitudinal studies showed that the social gradient was mainly explained by the path from work to health (“causal effect”) and not by the reverse pathway from health to work (“health selection effect”) [15, 16]. Our findings are restricted to two waves (2009 and 2013) for reasons of data availability. Furthermore, changes in job status may take time to affect HCU. Consequently, further studies are required considering a longer period of time to determine long-term or dynamic effects. While we examined changes in leadership positions in general due to power issues, future research might look at differences between industries. Furthermore, the reason for changes in leadership position (e.g., whether the change was compulsory or the individual elected to change voluntarily) should be analyzed in future studies.

## Conclusion

Findings of the present study emphasize the impact of leadership positions on the number of physician visits in men. Further studies are required to elucidate the underlying mechanisms (e.g., working conditions) between changes in leadership positions and physician visits in men.

## Abbreviations

BFI-S: Short version of the Big Five Inventory
DIW: Deutsches Institut für Wirtschaftsforschung
FE: Fixed effects
GSOEP: German Socio-Economic Panel
HCU: Health care use
